# Olfactory receptor 78 is expressed in hypothalamic vasopressin/oxytocin neurons, parenchymal microglia and choroidal macrophages in mice

**DOI:** 10.1186/s13041-022-00917-8

**Published:** 2022-04-04

**Authors:** Akiko Nakashima, Noriyuki Nakashima, Kie Nakashima, Makoto Takano

**Affiliations:** 1 Department of Physiology, Kurume University School of Medicine, 67 Asahi-machi, Kurume-shi, Fukuoka, 830-0011 Japan; 2 Laboratory of Developmental Neurobiology, Graduate School of Biostudies, Kyoto University, Yoshida Hon-machi, Kyoto, 606-8501 Japan

**Keywords:** Olfactory receptor 78, Central nervous system, Hypothalamic vasopressin, Oxytocin neurons, Choroid plexus, Vasculature, Macrophages, Microglia

## Abstract

Olfactory receptors have been detected in extraolfactory organs. Olfactory receptor 78 (Olfr78), proposed to respond to small organic acids, is widely expressed in the kidney, arterioles, colon, and prostate. However, its expression patterns in the brain remain largely unknown. Using immunohistochemistry, we revealed that Olfr78 was densely expressed in the hypothalamus and choroid plexus and sparsely expressed throughout the parenchyma. By costaining with cellular markers, we further found that Olfr78 was expressed in the somata and axons of vasopressin/oxytocin neurons in the hypothalamic paraventricular/supraoptic nuclei. Olfr78 was also strongly expressed in macrophages in the choroid plexus and moderately expressed in microglia near the parenchymal vasculature. Considering that these brain regions should communicate with cerebral blood flow, Olfr78 could contribute to sensing the humoral conditions surrounding the cerebrovascular system.

**Supplementary Information:**

The online version contains supplementary material available at 10.1186/s13041-022-00917-8.

## Background

Odourant receptors (ORs) constitute a superfamily of Gs protein-coupled receptors (GPCRs). Their extraolfactory expression has been intensively investigated and is increasingly reported. One olfactory receptor superfamily member, olfactory receptor 78 (Olfr78), is widely expressed, for example, in the brain [1] kidney [2], arterioles [3, 4], carotid body [5], macrophages [6], colon [7], and prostate [3].

Olfr78 is related to hypoxia-associated responses in the kidney and the carotid body [2, 5] and to bacterial metabolite sensing and hormone secretion in the colon [7]. In the prostate, Olfr78 is related to tumorigenesis and is also called prostate-specific GPCR (PSGR) [8]. Olfr78 is proposed to sense various metabolic byproducts of anaerobic cellular respiration or bacterial fermentation, such as short-chain fatty acids and lactate [9, 10]. Although its expression was originally detected in the brain [1], the localization of the Olfr78 protein in the brain remains largely uncharacterized [9] (Additional file 1: Fig. S1a–d). To determine the localization of Olfr78 in the brain at cellular resolution, we performed immunohistochemistry in mouse brain slices (for details, see the Additional file 1: Methods).

## Results

**Fig. 1:**
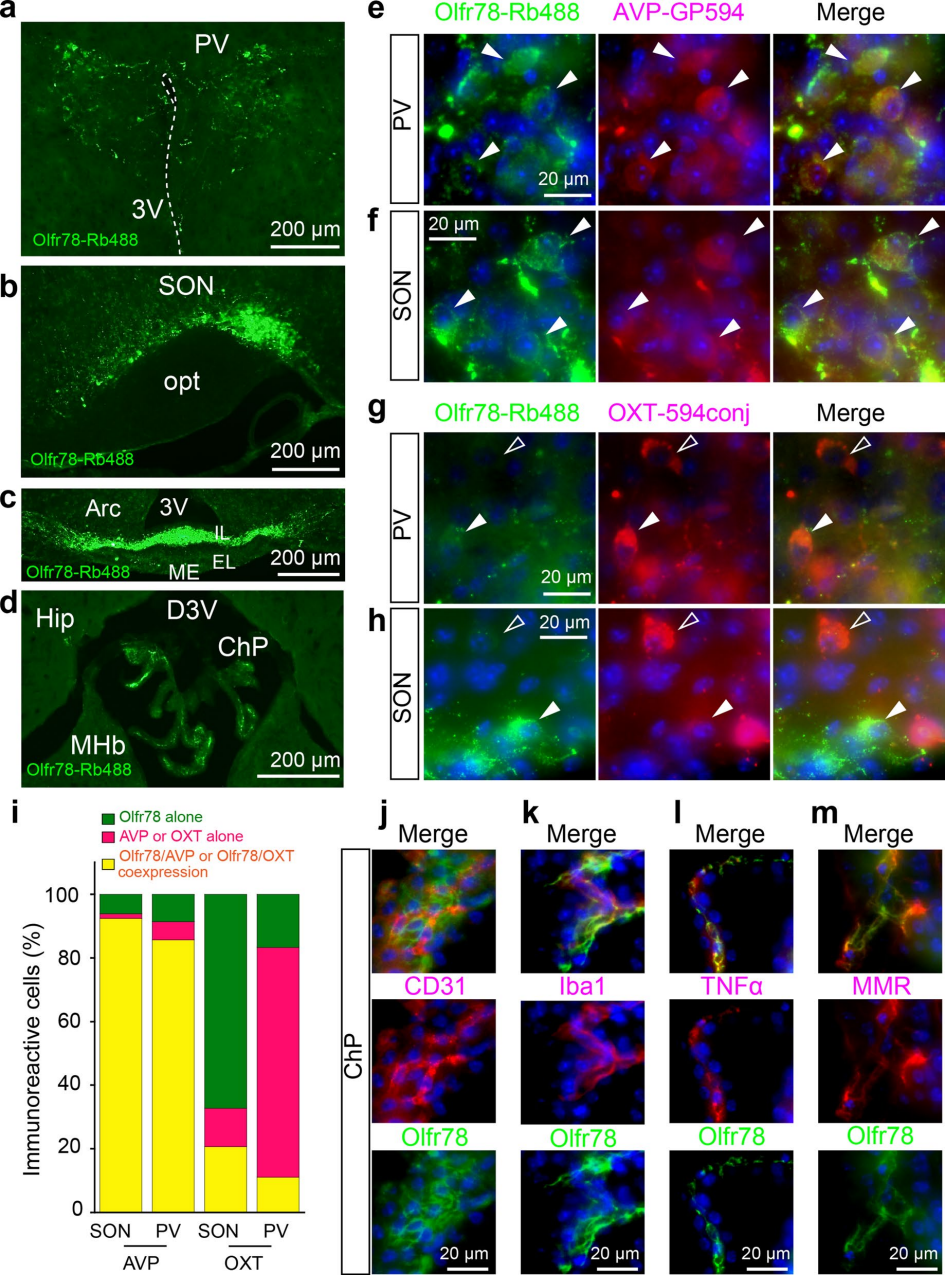
Olfr78 was expressed in AVP neurons in the hypothalamus. **a**–**d,** Olfr78 immunoreactivity (Olfr78-IR) was detected in the **a** paraventricular hypothalamus (PV), **b** supraoptic nucleus (SON) and **c** internal layer (IL) of the median eminence (ME) of the hypothalamus and in the **d** choroid plexus (ChP) by using an Alexa Fluor 488-conjugated anti-rabbit IgG secondary antibody (Olfr78-Rb488). **e**, **f** Olfr78-IR (green; Olfr78-Rb488) colocalization with arginine vasopressin (AVP)-IR in the **e** PV and **f** SON was detected by using an Alexa Fluor 594-conjugated anti-guinea pig IgG secondary antibody (red; AVP-GP594). **g**, **h** Olfr78-IR (green) colocalization with oxytocin (OXT)-IR (red) in the **g** PV and **h** SON was detected directly by using a DyLight 594-conjugated anti-OXT primary antibody (red; OXT-594conj). **i** Quantification of neurons expressing solely Olfr78, AVP or OXT or coexpressing Olfr78/AVP or Olfr78/OXT. n = 70 (PV) or 66 (SON) cells from 3 mice for AVR/Olfr78 and 72 (PV) or 58 (SON) cells from 3 mice for OXT/Olfr78. See Figure S4c for the means and standard deviations. **j**–**m** Olfr78-IR (green) was distinct from **j** CD31-IR in the endothelium and colocalized **k** with Iba1-IR in microglia/macrophages (red); **l** with TNFα-IR in M1 macrophages (red); and **m** with MMR-IR in M2 macrophages (red). Nuclei are coloured blue in (**e**–**h** and **j**–**m**). *3V* third ventricle; *opt*, optic tract, *EL* external layer, *Arc* arcuate hypothalamic nucleus, *Hip* hippocampus, *MHb* medial habenular nucleus, *D3V* dorsal third ventricle, *TNFα* tumour necrosis factor α, *MMR* macrophage mannose receptor

Olfr78 immunoreactivity (Olfr78-IR) was densely detected in the paraventricular region (PV), supraoptic nucleus (SON), and median eminence (ME) of the hypothalamus (Fig. 1a–c) and in the choroid plexus (Fig. 1d). In the hypothalamus, punctate Olfr78-IR was detected around somata and along neurites (Fig. 1e-h; Additional file 1: Fig. S2a–d). The PV and SON contain neuroendocrine neurons expressing arginine vasopressin (AVP) and oxytocin, whose neurites extend to the ME (Additional file 1: Fig. S3a–f). When costained with a guinea pig anti-AVP antibody, Olfr78-IR was detected in somata and axons of AVP-immunoreactive neurons (Fig. 1e-f, Additional file 1: Figs. S3a–c, S4a–d). The effective anti-oxytocin antibody was raised in rabbits, like the anti-Olfr78 antibody. Thus, after the initial anti-Olfr78 antibody reaction was enhanced by an Alexa Fluor 488-conjugated anti-rabbit IgG secondary antibody, the samples were incubated with the rabbit anti-oxytocin antibody directly conjugated to the fluorophore DyLight 594 (Lightning-Link, Abcam) (Fig. 1g-h, Additional file 1: Fig. S3e–f, S4d); in comparison, the fluorophore-conjugation method was evaluated by using another rabbit anti-AVP antibody (Additional file 1: Fig. S3b–c, S4a–b). Again, most AVP-IR was detected in Olfr78-immunoreactive cells with differential subcellular localization, confirming the efficacy of this method; in contrast, oxytocin-IR only partially overlapped with Olfr78-IR (Fig. 1e–i; Additional file 1: Figs. S3a–f, S4a–d). In the ME, Olfr78-IR was mainly located in the internal layer and sparsely in the external layer (Fig. 1c, Additional file 1: Fig. S4d).

In the choroid plexus, Olfr78-IR was detected in the stromal meshwork beneath the cuboidal epithelial cells of the papillary tip region (Fig. 1d, Additional file 1: Fig. S5a–d). The Olfr78-immunoreactive cells were adjacent to the CD31-immunoreactive vascular endothelium (Fig. 1j) and exhibited Iba1-IR, indicative of infiltrating stromal macrophages (Fig. 1k, Additional file 1: Fig. S5a). Olfr78-IR was detected in both types of macrophages: M1 macrophages with tumour necrosis factor α (TNFα)-IR (Fig. 1l, Additional file 1: Fig. S5b) and M2 macrophages with macrophage mannose receptor (MMR)-IR (Fig. 1m, Additional file 1: Fig. S5c).

Close observation also revealed that Olfr78-immunoreactive cells surrounded the vasculature (Additional file 1: Fig. S5e) in the parenchyma and exhibited Iba1-IR (Additional file 1: Fig. S5f), indicative of parenchymal microglia, consistent with the widespread detection of Olfr78 mRNA and protein expression throughout the brain (Additional file 1: Fig. S1a–d). Although astrocytes might express Olfr78 [11], no corresponding signals were confirmed in this study (Additional file 1: Fig. S5g).

## Discussion

Due to the technical limitations of available antibodies to detect oxytocin and Olfr78 simultaneously, we used a rabbit anti-oxytocin antibody directly conjugated to a fluorophore after enhancement of the weak Olfr78-IR with an anti-rabbit IgG secondary antibody. Despite concerns about the cross-reactivity of the secondary antibody, the fluorescence signals for Olfr78 and oxytocin were detected in distinct subcellular domains of the oxytocin-immunoreactive neurons (Fig. 1g-h, Additional file 1: Figs. S3d–e, S4d). Therefore, we concluded that at least some oxytocin neurons expressed Olfr78. Previous reports have demonstrated that Olfr78 is expressed in cells with chemosensory properties[3, 5, 6, 9]. Olfr78 responds to various small fatty acids, while the endogenous ligand for Olfr78 remains undetermined [4, 10, 12].

Olfr78-expressing fibres were detected in the internal layer of the ME, originating from magnocellular neurons in the SON/PVN and reaching the pituitary [13, 14] (Additional file 1: Fig. S4d). AVP neurons generate electrical signals in response to extracellular acidification induced by locally produced lactate under osmotic stress-induced hypoxia and should release AVP into the systemic circulation [15]. Olfr78 can be directly activated by lactate to increase AVP release in parallel to acidification-induced electrical activity [14, 15]. Notably, AVP/oxytocin secretion can be alternatively suppressed via cAMP/PKA cascades [16], in which Gs-coupled Olfr78 may participate [1]. Speculatively, Olfr78 might regulate the hormone release rate under fluctuating osmotic stress.

In the brain, both Iba1-immunoreactive parenchymal microglia and choroidal macrophages, which have different developmental origins [17], exhibited Olfr78-IR and were located near the vasculature. In bone marrow, Olfr78 in macrophages controls macrophage polarization towards the M1 or M2 phenotype [6]. In the choroidal stroma, both M1 and M2 macrophages displayed Olfr78-IR, which was stronger around the tip of the choroid plexus, suggesting that these choroidal macrophages could modulate the Olfr78 expression level during potential migration along the choroidal stroma. In the parenchyma, Olfr78-immunoreactive microglia with a sheath-like appearance apparently surrounded the cerebral vasculature. Therefore, Olfr78 in both microglia and macrophages can sense local metabolites influenced by surrounding humoral systems and might regulate the vasculature in response [3–5]. Global Olfr78 knockout leads to the dysfunction of cAMP-associated phenotypes, including hormone release, in different tissues [6, 7, 9], which could be under systemic feedback regulation. Conditional knockout based on the concomitantly expressed molecules could provide more specific insights into Olfr78 actions within the brain (Additional file 2).

In the central nervous system, the ME and choroid plexus are unique in passively or actively communicating with the vascular system across the blood–brain barrier. Indeed, our findings suggest that these Olfr78-expressing AVP/oxytocin neurons and microglia/macrophages could respond to metabolites [15, 16] from the vasculature, ventricle and parenchyma and potentially regulate cellular differentiation [6, 8] and cerebral blood flow [3–5].

## Supplementary Information

**Additional file 1.** Supplementary methods and figures.

**Additional file 2. **Datasheet containing the raw data presented in this study.

## Data Availability

All data generated or analysed during this study are included in this published article and its Additional files.

## Abbreviations

3V: Third ventricle
594Conj: Directly conjugated to DyLight 594
Arc: Arcuate hypothalamic nucleus
AVP: Arginine vasopressin
Cbl: Cerebellum
ChP: Choroid plexus
D3V: Dorsal third ventricle
EL: External layer
GAPDH: Glyceraldehyde-3-phosphate dehydrogenase
GP: Guinea pig
GPCR: Gs protein-coupled receptor
Hip: Hippocampus
Iba1: Ionized calcium-binding adapter molecule-1
IL: Internal layer
IR: Immunoreactivity
ME: Median eminence
MHb: Medial habenular nucleus
MMR: Macrophage mannose receptor
OE: Olfactory epithelium
Olfr78: Olfactory receptor 78
opt: Optic tract
OR: Odourant receptor
OXT: Oxytocin
PSGR: Prostate-specific G protein-coupled receptor
PV: Paraventricular hypothalamus
Rb: Rabbit
RT–PCR: Reverse transcription–PCR
SON: Supraoptic hypothalamic nucleus
TNFα: Tumour necrosis factor α
