# Supplementary material for: Olfactory receptor 78 is expressed in hypothalamic vasopressin/oxytocin neurons, parenchymal microglia and choroidal macrophages in mice
Source: Mol Brain. 2022 Apr 4;15:29. doi: 10.1186/s13041-022-00917-8 (PMC8981654; doi:10.1186/s13041-022-00917-8)
Supplement: Supplementary file 1 — Additional file 1. Supplementary methods and figures. [file 13041_2022_917_MOESM1_ESM.docx]

**Supplementary information**

**Olfactory receptor 78 is expressed in hypothalamic vasopressin/oxytocin neurons, parenchymal microglia and choroidal macrophages in mice**

**Akiko Nakashima** et al.,

Kurume University School of Medicine

**Materials and Methods**

**Treatment of animals**

We treated experimental animals in accordance with the Kurume University guidelines. C57BL/6 mice were purchased from SLC (Japan SLC, Inc., Shizuoka, Japan). The 8-week-old male mice used were anaesthetized by intraperitoneal injection of dexmedetomidine, midazolam and butorphanol (4, 10 and 0.5 mg/kg, respectively) before rapid decapitation with a sharp blade for extraction of mRNA or proteins or before fixation by perfusion with 4% paraformaldehyde solution (FUJIFILM Wako Pure Chemical Corporation, Osaka, Japan) for immunohistochemistry as described below.

**Cell culture**

HEK293T cells (ATCC, VA, USA) were cultured in Dulbecco’s modified Eagle’s medium (D-MEM; Wako Pure Chemical, Osaka, Japan) supplemented with 10% foetal bovine serum (FBS: Sigma–Aldrich, MO, USA) without antibiotics at 37°C and 5% CO_2_. The cells were passaged at 70% confluency.

**Reverse transcription–PCR (RT–PCR)**

Anaesthetized mice were decapitated, and tissues were excised by using tweezers and scissors. The respective areas of the brain were further dissected using a scalpel. Samples were dissolved in the appropriate solution for mRNA extraction using a kit (Roche, Basel, Switzerland). The mRNA of HEK293T cells was extracted similarly by using the same kit. cDNA was synthesized from mRNA using Superscript IV (Invitrogen, CA, USA). The sequence of the forward primer for detecting Olfr78 (NM_130866.4) was 5’-CCC TGG AGC ATC AGC TGG TAT ACC-3’; the sequence of the reverse primer was 5’- GAA AAA CAT CTG GGC AAG ACA GGC-3’. cDNA samples were subjected to RT–PCR with 35 cycles at 98°C for 10 s, 64°C for 20 s, and 72°C for 30 s using Advantage2 DNA polymerase (Takara Bio Inc., Shiga, Japan) and a thermal cycler (Eppendorf, Hamburg, Germany). The PCR products were electrophoresed with a loading dye (A3477 L, Toyobo, Osaka, Japan) and a ladder marker (Gene Ladder Wide 1, Nippon Gene Co., Ltd., Toyama, Japan) using a 2% agarose gel (A6013-500G, Sigma–Aldrich, MO, USA) by using an electrophoresis apparatus (i-MyRun. N, Cosmo Bio Co., Ltd., Tokyo, Japan). To confirm the nonexistence of Olfr78 cDNA in the HEK293T cells, we used actin beta cDNA (ACTB) as a positive control for the cDNA mixture by using the following primers: For the mouse ACTB brain cDNA; Forward, 5’-GCT GTA TTC CCC TCC ATC GT-3’: Reverse, 5’-AGT TTC ATG GAT GCC ACA GG-3’: For the human ACTB from HEK293T cells; Forward, 5’ GCC GTC TTC CCC TCC ATC GT 3’: Reverse, 5’ -AGT TTC GTG GAT GCC ACA GG-3’. The amplifying protocol was the same as above: 35 cycles at 98°C for 10 s, 64°C for 20 s, and 72°C for 30 s using Advantage2 DNA polymerase.

**Cloning of mouse Olfr78 cDNA**

We amplified mouse Olfr78 cDNA (NM_130866) from the mouse brain sample with the following primers: Forward, 5’- ACT ATA GGC TAG CCA CCA TGA GTT CCT GCA ACT TCA CC-3’: Reverse, 5’- AAT TCT CGA GTC ACG TGT TTC CCC CAG CTT-3’ with 35 cycles at 98°C for 10 s, 64°C for 20 s, and 72°C for 30 s using Advantage2 DNA polymerase (Takara Bio Inc., Shiga, Japan). The pCI mammalian expression vector (Promega, WI, USA) was linearized by PCR amplification by CloneAmp™ HiFi PCR Premix DNA polymerase (Takara Bio Inc.) with 35 cycles at 98°C for 10 s, 68°C for 180 s, and 72°C for 30 s using the following primers: Forward, 5’-AAA CAC GTG ACT CGA GAA TTC ACG CGT GGT-3’: Reverse, 5’- CAT GGT GGC TAG CCT ATA GTG AGT CGT ATT -3’. The PCR constructs were electrophoresed on a 0.8% agarose gel and purified with a Fast Gene Gel/PCR Extraction kit (NIPPON Genetics Co., Ltd., Tokyo, Japan). The purified PCR constructs were directly combined by the In-Fusion® HD Cloning Kit (Takara Bio Inc.) to yield the plasmid containing mouse Olfr78 cDNA (pCI-Olfr78). The plasmid was transferred into *E. coli* (Competent high DH5α, DNA-903, Toyobo) by transformation with incubation at 42°C for 30 s and on ice for 2 minutes. After further incubation with SOC medium at 37°C for 30 minutes, the transformed *E. coli* was plated in an LB agar plate (Agar Powder, Wako) containing 100 μg/ml ampicillin (Wako). The bacterial colonies selected by ampicillin were incubated at 37°C for 12 hours in 5 mL of LB medium containing 100 μg/ml ampicillin. The plasmid was extracted from the grown bacteria by NucleoSpin Plasmid EasyPure (MACHEREY-NAGEL, Düren, Germany). The Olfr78 cDNA sequence was validated (FASMAC Co., Ltd., Kanagawa, Japan) using the following primers: Forward, 5’-CTC CAC AGG TGT CCA CTC CC -3’ and Reverse, 5’- CAC TGC ATT CTA GTT GTG GTT TG -3’. To monitor the successful transfection of the Olfr78-exrepssing vector in the subsequent experiment, a gene cassette of the internal ribosome entry site with a green fluorescent protein (ires-GFP) was digested from pIRES2-EGFP (Clontech, CA, USA) at SalI/NotI restriction sites and inserted into the linearized pCI-Olfr78 vector at the SalI/NotI compatible sites using a ligation reagent (Ligation high Ver. 2, Takara Bio Inc.) with incubation at 16°C for 30 min to yield the Olfr78/GFP-coexpressing vector (pCI-Olfr78-ires-GFP). The pCI-Olfr78-ires-GFP plasmid was further amplified and purified as described above for pCI-Olfr78 for subsequent experiments.

**Heterologous expression system**

HEK293T cells (ATCC, VA, USA) were plated on coverslips and cultured in Dulbecco’s modified Eagle’s medium (D-MEM; Wako Pure Chemical, Osaka, Japan) supplemented with 10% foetal bovine serum (FBS: Sigma–Aldrich, MO, USA) without antibiotics at 37°C and 5% CO_2_. HEK293T cells were transfected with 1 µg of pCI-Olfr78-ires-GFP or or pEGFP-N1 (a negative control; Clontech) using Lipofectamine 2000 (Thermo Fisher Scientific, MA, USA) at 50-60% confluency in D-MEM with 10% FBS. After transfection, HEK293T cells were incubated for 24 h prior to immunocytochemistry.

Western blotting

After mice were decapitated, tissue samples were dissolved in Laemmli sample buffer (Bio–Rad Laboratories, CA, USA) at 25°C supplemented with 10% 2-mercaptoethanol (Sigma–Aldrich) and were then heated at 95°C for 5 min and used for subsequent western blotting. Samples (10 µL) were electrophoresed using a precast 10% Mini-PROTEAN TGX gel (Bio–Rad Laboratories) in a Mini-PROTEAN Tetra Cell electrophoresis system (Bio–Rad Laboratories) according to the manufacturer’s instructions with a protein size marker (Precision Plus Protein Dual Color Standards, Bio–Rad Laboratories). Similarly, total proteins from HEK293T cells were collected as a negative control for detecting Olfr78. Then, proteins were transferred to PVDF membranes (Midi Format 0.2 µm PVDF, Cat. #1704157, Bio–Rad Laboratories) using a Trans-Blot Turbo Transfer System (Bio–Rad Laboratories) for 7 min at 25 V. The PVDF membranes were sequentially incubated overnight at 4°C with primary antibodies against Olfr78 (NLS6332, lot# 39563; Novus Biologicals, CO, USA)[1] and glyceraldehyde-3-phosphate dehydrogenase (GAPDH; M171.3, clone# 3H12, lot# 006, MBL Co. Ltd, Tokyo, Japan) (all 1:1000) diluted with Can Get Signal immunoreaction enhancer solution (NKB-101, lot# 142500, Toyobo), washed with Tris-buffered saline containing 0.1% Tween (TBST: 10 mM Tris, 150 mM NaCl; pH 7.6) 3 times for 10 min each and incubated overnight at 4°C. The membranes were incubated with a horseradish peroxidase (HRP)-conjugated anti-rabbit IgG secondary antibody (7074P2, Cell Signaling Technology) or an HRP-conjugated anti-mouse IgG secondary antibody (ab6823, Abcam, Cambridge, UK) at a 1:3000 dilution for 1.5 h at 25°C and were then washed with TBST. Then, the membranes were washed again carefully. IR was detected using ECL Prime Western Blotting Detection Reagent (RPN2232, Cytiva, NJ, USA). Images were acquired using an Amersham Imager 600 (GE Healthcare Biosciences, NJ, USA).

**Fluorescence immunohistochemistry**

The anaesthetized mice were fixed by perfusion with 4% paraformaldehyde in phosphate-buffered saline (PBS). Tissues were postfixed for 10 h, cryoprotected by overnight incubation in PBS containing 30% w/v sucrose at 4°C, mounted in OCT Embedding Compound (Sakura Finetek, Tokyo, Japan) and frontally sectioned at a 30-μm thickness using a cryostat (CM3050S, Leica Microsystems, Wetzlar, Germany) at -20°C. Sections were then incubated at 25°C overnight in an appropriate blocking solution containing the following primary antibodies: rabbit anti-Olfr78 (NLS6332, lot# 39563; Novus Biologicals) [1], rabbit anti-AVP (ab213708, lot# GR3278819, Abcam) [2], guinea pig anti-AVP (403004, lot# 1-4, Synaptic Systems, Göttingen, Germany) [3], rabbit anti-oxytocin (ab212193, lot# GR3227039-5, Abcam) [4], chicken anti-vimentin (AB5733, lot# 3474064, Merck KGaA, Darmstadt, Germany) [5], mouse anti-Iba1 (GTX632426: clone ID GT10312, lot# 44307; GeneTex, CA, USA) [6], mouse anti-TNFα (60291-1-Ig: clone ID 7B8A11, lot# 10004304, Proteintech, IL, USA) [7], mouse anti-CD206 (60143-1-Ig: clone ID 2A6A10, lot# 10021878; Proteintech) [8] and chicken anti-Glial fibrillary acidic protein (NBP1-05198, lot# 7529-4, Novus Biologicals) [9]. The samples were washed with PBS and incubated in PBS with Triton X-100 (FUJIFILM Wako Pure Chemical Corporation), 4',6-diamidino-2-phenylindole (DAPI, diluted 1:1000; Nacalai tesque, Kyoto, Japan) and the appropriate anti-IgG secondary antibodies (diluted 1:200; Alexa Fluor 488-conjugated anti-rabbit, ab150061; Alexa Fluor 594-conjugated anti-rabbit, ab150064; Alexa Fluor 488-conjugated anti-rat, ab150153; DyLight 594-conjugated anti-guinea pig, ab9696; Alexa Fluor 488-conjugated anti-mouse, ab150109 (all from Abcam); Alexa Fluor 594-conjugated anti-chicken, 703-585-155, Jackson ImmunoResearch Europe, Ltd, Cambridge, UK) for 1.5-2 h, washed in PBS, mounted onto MAS-coated glass slides (Matsunami Glass, Tokyo, Japan), coverslipped using Vectashield antifade reagent (Vector Labs, CA, USA) and tightly sealed. The above procedures were conducted at 25°C. In some experiments where the primary antibodies raised in rabbits were unavoidably used, we first applied the rabbit anti-Olfr78 antibody, whose signal was enhanced by the Alexa Fluor 488-conjugated donkey anti-rabbit IgG secondary antibody (ab150061, Abcam). Samples were washed in PBS at 25°C for 24 h and were then incubated with the anti-AVP antibody (ab213708, Abcam) or anti-oxytocin antibody (ab212193, Abcam), both of which were directly conjugated to DyLight 594 by using a fluorophore conjugation kit following the manufacturer’s instructions (Lightning-Link, ab201801, Abcam). The anti-Olfr78 antibody directly conjugated to DyLight 488 (Lightning-Link, ab201799, Abcam) was insufficient to yield detectable fluorescent signals when used alone (Figure S2c); thus, it was required to use the anti-rabbit IgG secondary antibody to detect Olfr78 in this study. For immunocytochemistry on HEK293T cells, the transfected HEK293T cells cultured on coverslips were immersion-fixed in ice-cold 4% PFA for 15 min, washed three times with PBS for more than 30 min, incubated with the rabbit anti-Olfr78 primary antibody (NLS6332, Novus Biologicals) at 25°C for 6 h, washed three times with PBS and incubated with an Alexa Fluor 594 conjugated anti-rabbit secondary antibody at 25°C for 1 h. Then, the coverslips were washed with distilled water, dried, mounted on a slide (Matsunami Glass Ind., Ltd., Osaka, Japan) with Vectashield antifade reagent (Vector Labs, CA, USA) and tightly sealed. Fluorescence signals were detected using a fluorescence microscope (BX50, Olympus, Tokyo Japan), imaged with a digital camera (DP72, Olympus) and analysed with cellSens image analysis software (Olympus). The following antibody combination yielded no apparent immunoreactive signals in the protocol described above at a maximum dilution of 1:50: anti-oxytocin goat IgG (SAB2501950, lot# 9023G2, Sigma, USA) [10] enhanced by Alexa Fluor 488-conjugated anti-goat IgG (ab150129, Abcam) (Figure S3f). The intensity of the images was measured by an image processing program (ImageJ 1.53k, NIH, USA).

**Statistical analysis**

Statistical analysis was performed with KaleidaGraph 4 (Synergy Software, PA, USA).

**
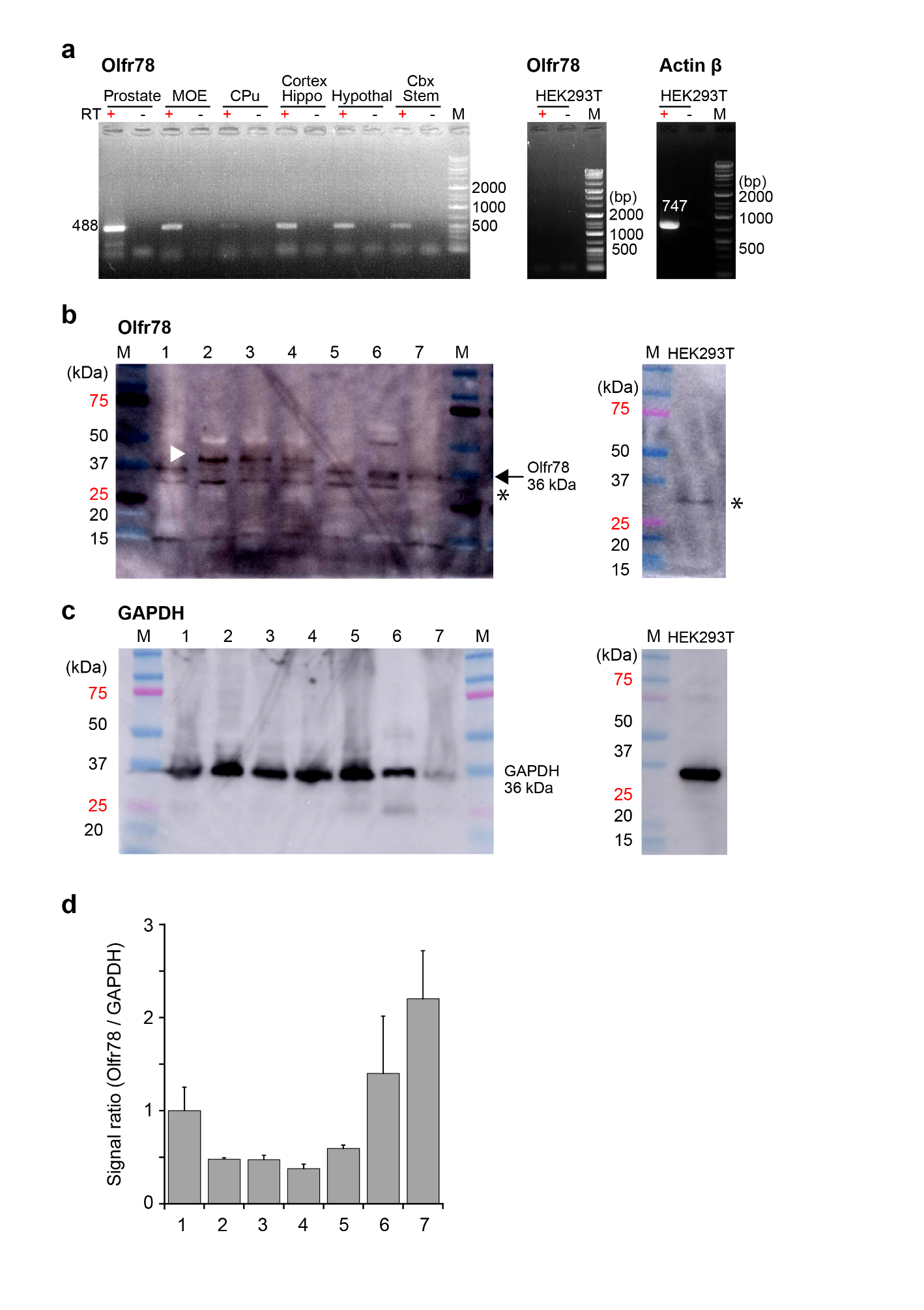
**

**Figure S1**

**a,** Olfactory receptor 78 (Olfr78, NM_130866.4) cDNA was detected as a band of 488 base pairs (bp). RT(+) and RT(-) indicate amplification with or without reverse transcription of mRNA, respectively. Prostate glands (prostate) and olfactory epithelium (OE) were used as positive controls. The striatum, cortex, hippocampus (Hip), hypothalamus (Hypothal), and cerebellum (Cbl) exhibited the signal. The mRNA of mouse Olfr78 was not detected in HEK293T cells by RT–PCR, indicating that HEK293T cells worked as a negative control. **b, c,** Western blotting for Olfr78 (36 kDa) using tissue extracts from various regions of the brain. Representative original gels used for western blotting for (**b**) Olfr78 (exposed for 1 s) and (**c**) glyceraldehyde-3-phosphate dehydrogenase (GAPDH, 36 kDa; exposed for 0.1 s). Lanes: 1, olfactory epithelium; 2, cortex and hippocampus; 3, striatum, thalamus and hypothalamus; 4 cerebellum and brain stem; 5, prostate gland; 6, lung; and 7, kidney. Lanes 1, 5, 6 and 7 were positive controls. The mouse Olfr78 protein was not detected at 36 kDa in HEK293T cells by western blotting. A band with a lower molecular weight of approximately 30 kDa (asterisk) was detected in HEK293T cells, indicating that this band was a nonspecific signal. Higher molecular weight bands above 37 kDa (white arrowhead) were consistently observed in 4 independent experiments, indicating that Olfr78 in the brain (lanes 2, 3 and 4) may undergo modifications, including forming complexes with other molecules. ORs frequently appear as multiple bands in western blots[11]. However, the identity of the band remained unknown. Thus, this band was excluded from the subsequent quantification. **d**, Relative Olfr78 expression (36 kDa band in **b**) compared to total GAPDH expression (36 kDa band in **c**); the ratios were further normalized to those in the olfactory epithelium (lane 1). n = 3 independent experiments. Mean ± SEM.

**
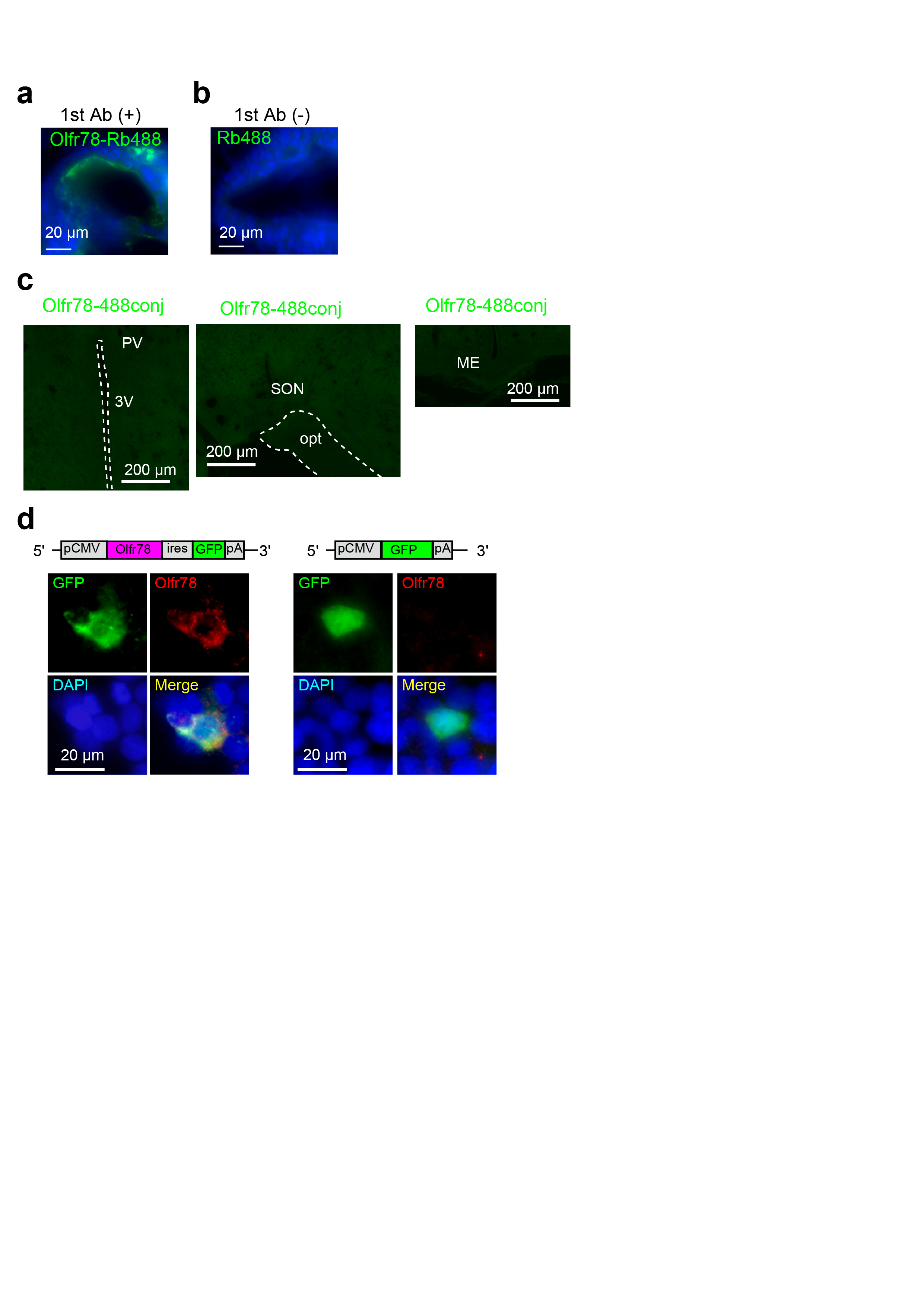
**

**Figure S2**

Validation of the available antibody against Olfr78 (NLS6332, Novus Biologicals) for immunohistochemistry was confirmed in the mouse prostate gland. **a, b,** Olfr78 immunoreactivity detected by the Alexa Fluor 488-conjugated anti-rabbit IgG secondary antibody in the presence (**a**) and absence (**b**) of the primary antibody (1^st^ Ab; Olfr78-Rb488 and Rb488, respectively). Nuclei are coloured blue. **c,** The anti-Olfr78 antibody (NLS6332, Novus Biologicals) directly conjugated to DyLight 488 (Lightning-Link, Abcam) (Olfr78-488conj) showed no apparent fluorescence signals in the PV, SON and ME when used alone. Therefore, the anti-Olfr78 antibody used in this study needed enhancement by reaction with the Alexa Fluor 488-conjugated anti-rabbit IgG secondary antibody, as shown in the brain in Figure 1 and in all subsequent experiments. **d,** The efficacy of the anti-Olfr78 antibody was also confirmed by immunohistochemistry in HEK293T cells exogenously expressing Olfr78/GFP in comparison to HEK293T cells expressing GFP only (a negative control). A vector to introduce Olfr78/GFP cDNAs joined by the internal ribosome entry site (ires) and a control vector expressing only GFP are described above. **Abbreviations:** PV, paraventricular hypothalamus; 3V, third ventricle; SON, supraoptic nucleus; opt, optic tract; ME, median eminence; HEK293T, human embryonic kidney 293T: GAPDH, glyceraldehyde-3-phosphate dehydrogenase. pCMV, cytomegalovirus promoter. pA, polyadenylation signal: GFP, green fluorescent protein: DAPI, 4',6-diamidino-2-phenylindole.

**
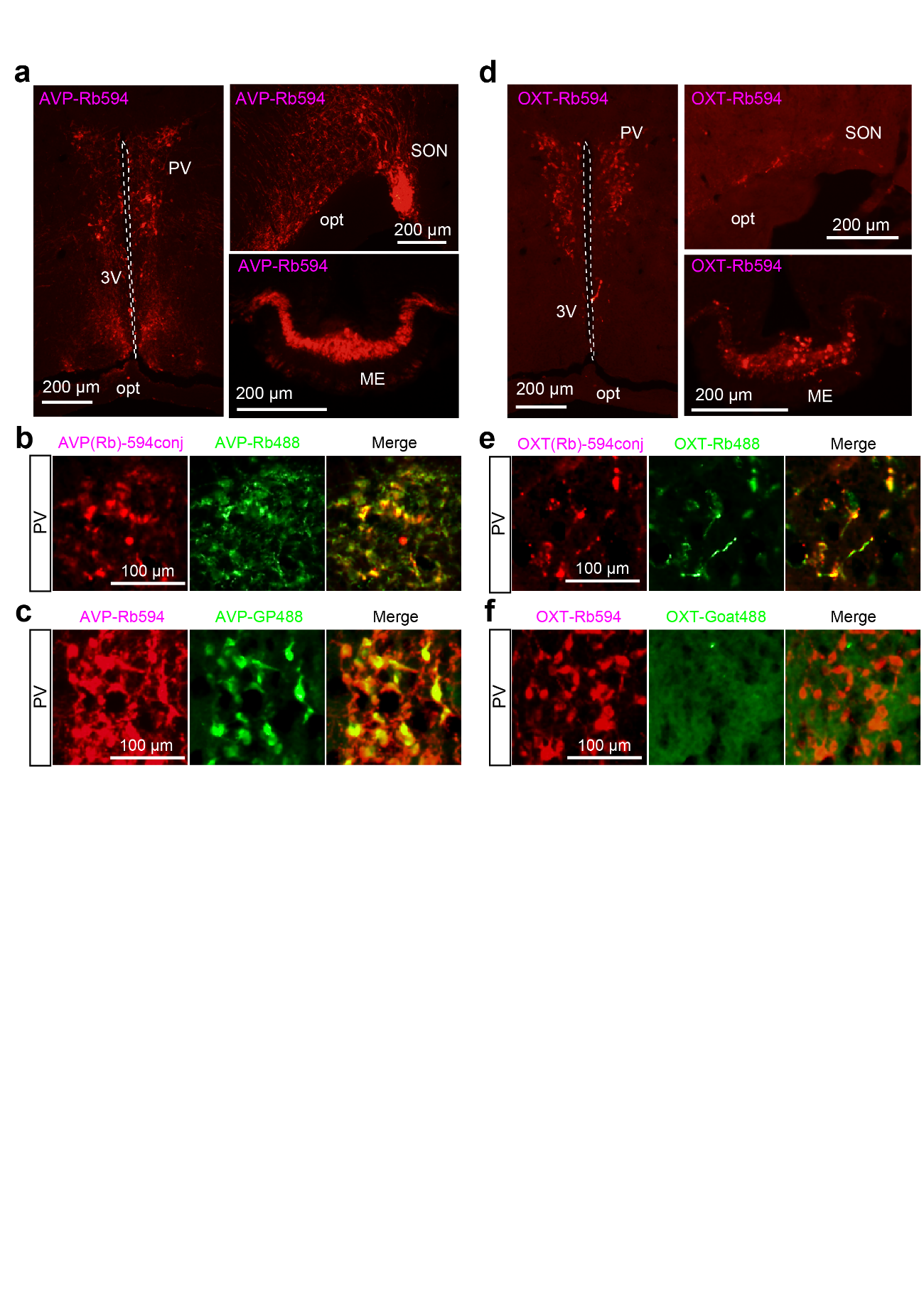
**

**Figure S3**

Validation of available antibodies against arginine vasopressin (AVP) and oxytocin (OXT) in the hypothalamus. Neurons with AVP-IR and OXT-IR were clustered in the paraventricular region (PV), supraoptic nucleus (SON) and median eminence (ME). opt, optic tract; 3V, third ventricle. **a,** Immunoreactive signals produced by the rabbit anti-AVP antibody (ab213708, Abcam) enhanced by the Alexa Fluor 594-conjugated anti-rabbit IgG secondary antibody (AVP-Rb594). **b,** The signal of the rabbit anti-AVP antibody detected by direct conjugation of DyLight 594 (Lightning-Link, Abcam) (red; AVP(Rb)-594conj) was comparable to the signal additionally enhanced by the Alexa Fluor 488-conjugated anti-rabbit secondary antibody (green; AVP-Rb488) in the soma. Thus, the direct dye conjugation method was effective for this antibody. **c,** The guinea pig anti-AVP antibody (green; AVP-GP488, 403004, Synaptic Systems) showed a signal comparable to that of the rabbit anti-AVP antibody (red; AVP-Rb594). **d,** Rabbit anti-OXT antibody (ab212193, Abcam) with enhancement by the Alexa Fluor 594-conjugated anti-rabbit IgG secondary antibody (OXT-Rb594). **e,** The signal of the rabbit anti-OXT antibody detected by direct conjugation of DyLight 594 (red; OXT(Rb)-594-conj) was comparable to that of the signal enhanced by the Alexa Fluor 488-conjugated anti-rabbit IgG secondary antibody (green; OXT-Rb488) in the soma. Thus, the direct dye conjugation method was effective for this antibody. **f,** The goat anti-OXT antibody (SAB2501950, Sigma) showed no apparent signal even after enhancement by the Alexa Fluor 488-conjugated anti-goat IgG secondary antibody (green; OXT-Goat488), unlike the rabbit anti-OXT antibody (red; OXT-Rb594). Thus, the rabbit anti-OXT antibody was the only antibody available in this study to detect oxytocin expression. Note that the dense spots of (**a**) AVP-IR and (**d**) OXT-IR were assumed to be axon swellings[12].

**
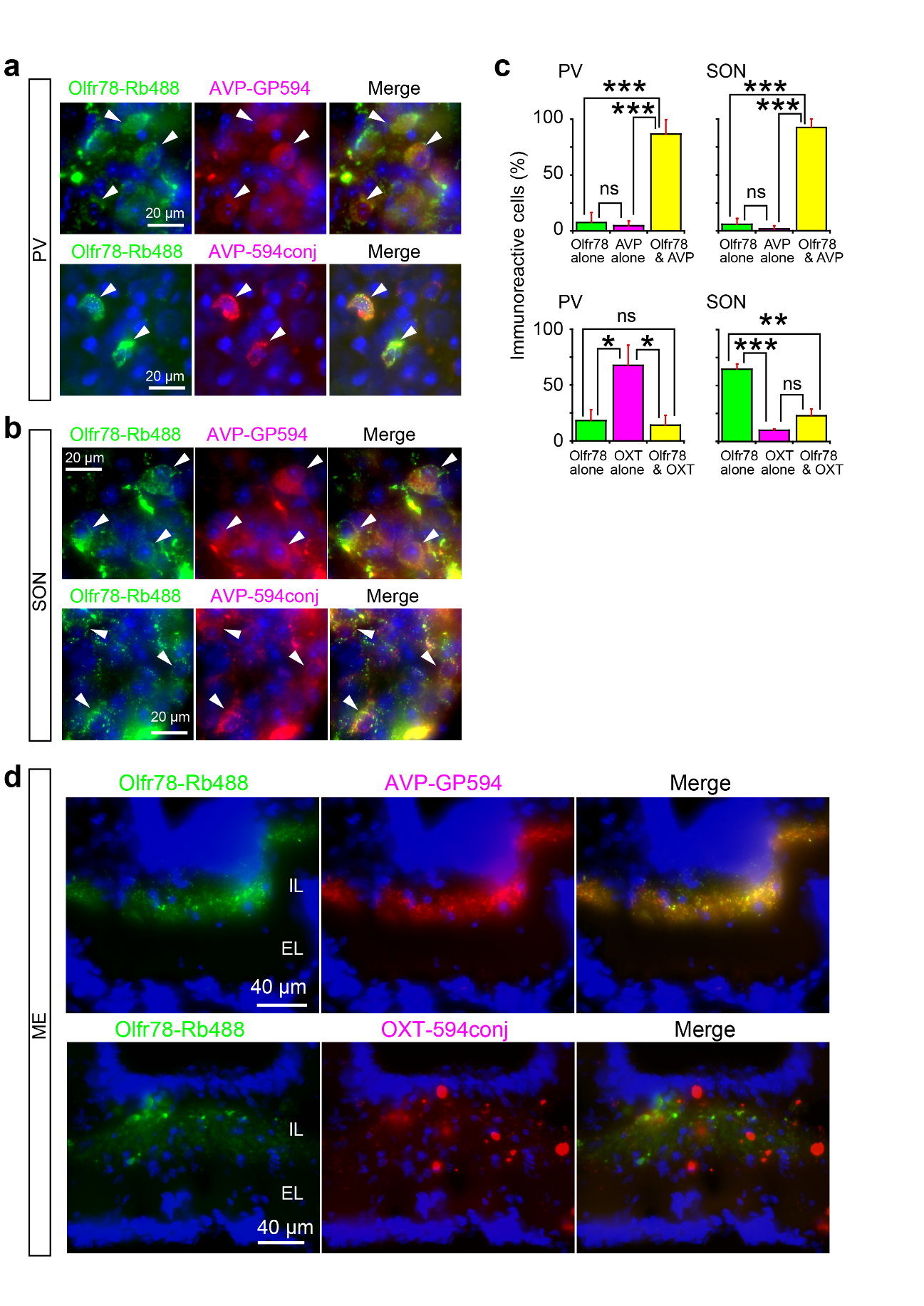
**

**Figure S4**

Subcellular localization of Olfr78 immunoreactivity (Olfr78-IR) in arginine vasopressin (AVP) and oxytocin (OXT) neurons. **a, b,** Olfr78-IR (green; Olfr78-Rb488) was detected in the somata of AVP neurons using guinea pig anti-AVP antibody enhancement by Alexa Fluor 594-conjugated anti-guinea pig IgG secondary antibody (red; AVP-GP594, upper panels). Additionally, Olfr78-IR (green) was detected in the somata of AVP neurons using the DyLight 594-conjugated rabbit anti-AVP antibody (Lightning-Link, Abcam) (red; AVP-594conj, lower panels) in the (**a**) paraventricular hypothalamus (PV) and (**b**) supraoptic nucleus (SON). The filled triangles indicate cells with both Olfr78-IR and AVP-IR. **c**, Quantification of cells with Olfr78-IR, AVP-IR or both Olfr78-/AVP-IR (upper panels) or Olfr78-IR, OXT-IR, or both Olfr78-/OXT-IR (lower panels) in the PV and SON was determined from three mice each. Mean ± SD. The cells that exhibited immunoreactive signals more than 4 standard deviations above the mean background signal (in five background areas) were counted. **d,** Olfr78-IR, AVP-IR and OXT-IR in the median eminence (ME). In the ME, Olfr78-IR was mainly detected in the internal layer (IL) compared to the external layer (EL), indicating that these terminals originate from magnocellular neurons in the PVN and SON[13],[14]. Olfr78-IR colocalized mostly with secondary antibody-enhanced AVP-IR (AVP-GP594) but less with DyLight 594-conjugated OXT-IR (Lightning-Link, Abcam) (OXT-594conj). Thus, OXT-IR was assessed only in the SON and PV in (**c**). The filled arrowheads indicate areas of colocalized IR. Nuclei are coloured blue in (**a-b** and **d**).

**Statistics for Figure S4c.**

Upper left panel (Olfr78 vs. AVP in PV). One-way ANOVA; F(2, 6) = 73.78, P < 0.0001. Upper right panel (Olfr78 vs. AVP in SON). One-way ANOVA; F(2, 6) = 258.89, P < 0.0001. Lower left panel (Olfr78 vs. OXT in PV). One-way ANOVA; F(2, 6) =16.11, P = 0.0039. Lower right panel (Olfr78 vs. OXT in SON). One-way ANOVA; F(2, 6) = 82.60, P < 0.0001. *Post hoc* Bonferroni test: Significant; *, P < 0.01; **, P < 0.001 ***; P < 0.0001. ns, nonsignificant.

**
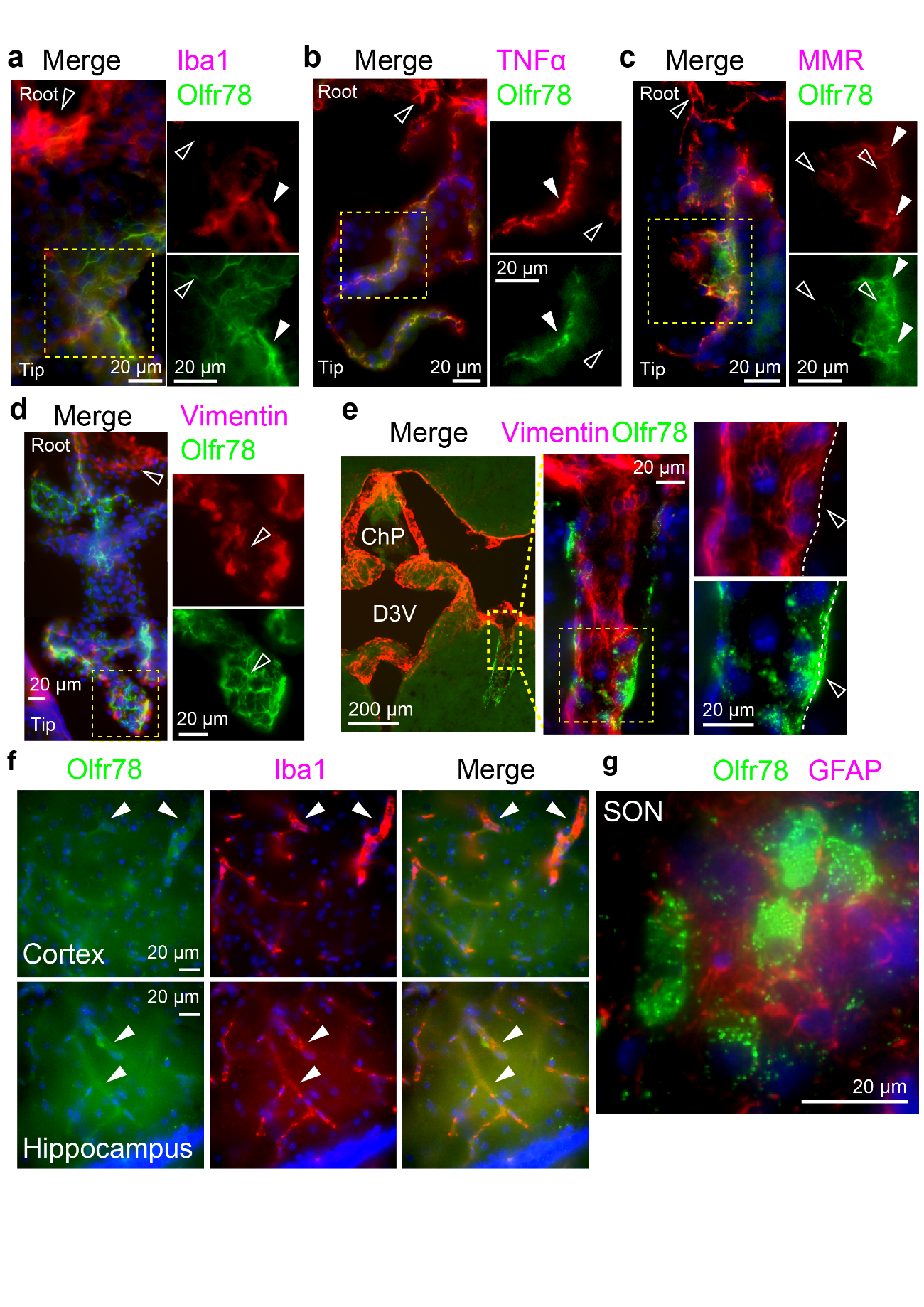
**

**Figure S5**

**a-c,** Colocalization and noncolocalization of (**a**) Iba1-IR, (**b**) TNFα-IR and (**c**) MMR-IR with Olfr78-IR in the choroid plexus. Olfr78-IR was weak in the root and strong in the papillary tip of the choroid plexus (**a-c**; Merge), indicating that there was a gradient in the expression level of Olfr78 within the population of choroidal macrophages. **d-e,** Olfr78-IR was detected as distinct fluorescent foci near the vascular smooth muscle with vimentin-IR in the (**d**) choroid plexus and (**e**) parenchyma. The white dotted line indicates the boundary between vimentin-IR and Olfr78-IR in (**e**). **f,** Olfr78-IR was sparsely detected in the parenchyma, which showed a sheath-like appearance with microglial Iba1-IR in the cortex and hippocampus. **g,** Olfr78-IR in the SON was detected in spindle-shaped cells surrounded by astrocytes with glial fibrillary acidic protein (GFAP)-IR. The filled and open arrowheads indicate colocalized and noncolocalized immunoreactive signals, respectively. The yellow dotted squares are magnified rightward in (**a-e**). Nuclei are coloured blue in (**a-g**). **Abbreviations:** D3V, dorsal third ventricle; SON, supraoptic nucleus.

**References for Supplementary Information**

1. Cao W, Li F, Yao J, Yu J. Prostate specific G protein coupled receptor is associated with prostate cancer prognosis and affects cancer cell proliferation and invasion. BMC Cancer. 2015;15:915.

2. Crane-Smith Z, Schoenebeck J, Graham KA, Devenney PS, Rose L, Ditzell M, et al. A Highly Conserved Shh Enhancer Coordinates Hypothalamic and Craniofacial Development. Front Cell Dev Biol. 2021;9.

3. Barad Z, Jacob-Tomas S, Sobrero A, Lean G, Hicks AI, Yang J, et al. Unique organization of actin cytoskeleton in magnocellular vasopressin neurons in normal conditions and in response to salt-loading. eNeuro. 2020;7:1–13.

4. Yuan XS, Wei HH, Xu W, Wang L, Qu WM, Li RX, et al. Whole-brain monosynaptic afferent projections to the cholecystokinin neurons of the suprachiasmatic nucleus. Front Neurosci. 2018;12:1–13.

5. Parkash J, Messina A, Langlet F, Cimino I, Loyens A, Mazur D, et al. Semaphorin7A regulates neuroglial plasticity in the adult hypothalamic median eminence. Nat Commun. 2015;6.

6. Liu D, Bai X, Ma W, Xin D, Chu X, Yuan H, et al. Purmorphamine Attenuates Neuro-Inflammation and Synaptic Impairments After Hypoxic-Ischemic Injury in Neonatal Mice via Shh Signaling. Front Pharmacol. 2020;11:1–15.

7. Zhao H, Wang Y, Li B, Zheng T, Liu X, Hu BH, et al. Role of Endoplasmic Reticulum Stress in Otitis Media. Front Genet. 2020;11:1–11.

8. Tao H, Zhao H, Ge D, Liao J, Shao L, Mo A, et al. Necroptosis in pulmonary macrophages promotes silica-induced inflammation and interstitial fibrosis in mice. Toxicol Lett. 2022;355:150–9.

9. Khan SS, Sobu Y, Dhekne HS, Tonelli F, Berndsen K, Alessi DR, et al. Pathogenic lrrk2 control of primary cilia and hedgehog signaling in neurons and astrocytes of mouse brain. Elife. 2021;10:1–25.

10. Tarquis-Medina M, Scheibner K, González-García I, Bastidas-Ponce A, Sterr M, Jaki J, et al. Synaptotagmin-13 is a neuroendocrine marker in brain, intestine and pancreas. Int J Mol Sci. 2021;22.

11. Shepard BD, Natarajan N, Protzko RJ, Acres OW, Pluznick JL. A cleavable N-terminal signal peptide promotes widespread olfactory receptor surface expression in HEK293T cells. Meyerhof W, editor. PLoS One. 2013;8:e68758.

12. Russell JA. Fifty Years of Advances in Neuroendocrinology. Brain Neurosci Adv. 2018;2:2398212818812014.

13. Burbach JPH, Luckman SM, Murphy D, Gainer H. Gene regulation in the magnocellular hypothalamo-neurohypophysial system. Physiol Rev. 2001;81:1197–267.

14. Kawakami N, Otubo A, Maejima S, Talukder AH, Satoh K, Oti T, et al. Variation of pro-vasopressin processing in parvocellular and magnocellular neurons in the paraventricular nucleus of the hypothalamus: Evidence from the vasopressin-related glycopeptide copeptin. J Comp Neurol. 2021;529:1372–90.
